# 1662. Impact of a Documented Penicillin Allergy on Antibiotic Selection in Patients with Osteomyelitis

**DOI:** 10.1093/ofid/ofac492.128

**Published:** 2022-12-15

**Authors:** Caitlin Naureckas Li, Katherine Herman, Ramy Yim, Mari M Nakamura, Esther Chu, Jayme Wilder, Maria Alfieri, Benjamin Ethier, Brittany Esty

**Affiliations:** Boston Children's Hospital, Riverside, Illinois; Boston Children’s Hospital, Boston, Massachusetts; Boston Children's Hospital, Riverside, Illinois; Boston Children's Hospital/Harvard Medical School, Boston, Massachusetts; Boston Children's Hospital, Riverside, Illinois; Boston Children's Hospital, Riverside, Illinois; Boston Children's Hospital, Riverside, Illinois; Boston Children's Hospital, Riverside, Illinois; Boston Children's Hospital, Riverside, Illinois

## Abstract

**Background:**

Penicillin allergy is the most commonly reported drug allergy, affecting approximately 10% of patients. Although historically there has been concern about administering cephalosporins to patients allergic to penicillin, the rates of cross-reactivity are only approximately 2%. As cephalosporins are the first-line and safest treatment for many infections, unnecessary avoidance of cephalosporins places patients at risk of poor disease outcomes and antibiotic-associated harms. We assessed the relationship between penicillin allergy label and antibiotic selection in pediatric patients with acute osteomyelitis.

**Methods:**

We performed a retrospective review of inpatients at our quaternary children’s hospital diagnosed with osteomyelitis between 2011 and 2021. During this period, the institutional osteomyelitis clinical pathway recommended clindamycin for patients with cephalosporin but not penicillin allergy. We compared rates of antibiotics used as definitive therapy between patients with and without documented penicillin allergy.

**Results:**

Of 365 patients hospitalized with a diagnosis of osteomyelitis, 41 (11.2%) had a documented penicillin allergy. First-generation cephalosporins were administered less frequently to penicillin-allergic patients compared with those without documented penicillin allergy (37% vs. 58%, p=.009), while clindamycin was administered more frequently (22% vs. 10%, p=.02). There was no significant difference in vancomycin use (2% vs. 9%, p=.17).

**Conclusion:**

Patients with osteomyelitis and a penicillin allergy label were significantly less likely to receive a first-generation cephalosporin and significantly more likely to receive clindamycin. Given higher regional resistance rates and more frequent adverse effects, including *Clostridioides difficile* infection, of clindamycin relative to first-generation cephalosporins, these data support the need for quality improvement work to increase cephalosporin use in patients with penicillin allergy labels.

**Disclosures:**

**Mari M. Nakamura, MD, MPH**, Gilead: Grant/Research Support.

